# Clinical evidence and biological considerations in the clinical use of lecanemab – experience at an academic medical center

**DOI:** 10.1002/alz70858_107460

**Published:** 2025-12-26

**Authors:** Lawrence S. Honig, Wendy P. Gonzalez, Jee Min Kim, Vanessa DiMuro, Karen L. Bell, Radhika Jagannathan, James M. Noble, Karen Marder, Richard Mayeux

**Affiliations:** ^1^ Columbia University Irving Medical Center, New York, NY, USA

## Abstract

**Background:**

Lecanemab and donanemab are anti‐amyloid antibodies proven in 18‐month clinical trials to slow progression of early Alzheimer's disease by biomarker, cognitive and functional measures. While both are now approved in many countries, lecanemab was first USA‐approved in January2023, and Medicare‐covered since July2023. Early‐AD patients are receiving therapy, but drug access has been limited by barriers, many imposed by use recommendations, and by insurer‐payor criteria. These partly reflect attempts to apply clinical trial exclusions to early AD patients, and concerns over adverse‐effect rates that might occur with widespread use.

**Method:**

We report, with IRB‐approval, our practice experience, prescribing on‐label, without other limitations, in >190 diverse patients, age 35‐90, using various infusion and MRI facilities. Patients all had amyloid confirmation by CSF (72%), amyloid‐PET (15%), or both (14%); 89% have accepted optional APOE‐genotyping, with 41% non‐carriers, 46% E4‐heterozygotes, and 13% E4‐homozygotes.

**Result:**

Patients have had 1‐40 biweekly infusions. Adverse event rates have been similar to those in clinical trials with 15% infusion reactions (headache, chills, warmth, fatigue, pruritus), usually only at first 1‐3 infusions. Overall rate of ARIA–E has been 10.6% (mostly in first 3 months), with 2 of 20 cases recurrent, and with a lower incidence of ARIA‐H. Only 2 patients (1%) have had symptomatic ARIA, both ARIA‐E, an E4 heterozygote with cortical visual loss, and an E4 homozygote with aphasia, both with concurrent mild ARIA‐H. The latter individual unfortunately died during ICU treatment for refractory focal status epilepticus; this was the only death. Most ARIA cases have resumed infusions after resolution; overall, there was discontinuation of therapy in 9% of treated patients (4% due to ARIA, 2% due to persistent infusion reactions, 3% due to disinterest).

**Conclusion:**

Our single‐site findings suggest that “real‐world” experience with lecanemab may be not dissimilar to that in clinical trials, and that despite a small risk of unavoidable serious adverse events, there can be ease of administration and MRI procedures, good tolerability, and relative safety. It is appropriate to use extant clinical evidence, and biological plausibility, rather than provide barriers to access, in discussing potential risks and benefits, and providing access to patients and their families.

